# Evaluation of the taxonomic status of populations assigned to *Phyllomedusa hypochondrialis* (Anura, Hylidae, Phyllomedusinae) based on molecular, chromosomal, and morphological approach

**DOI:** 10.1186/1471-2156-14-70

**Published:** 2013-08-12

**Authors:** Daniel Pacheco Bruschi, Carmen Sílvia Busin, Luís Felipe Toledo, Gilda Andrade Vasconcellos, Christine Strussmann, Luiz Norberto Weber, Albertina Pimentel Lima, Jucivaldo Dias Lima, Shirlei Maria Recco-Pimentel

**Affiliations:** 1 Departamento de Biologia Estrutural e Funcional, Instituto de Biologia, Universidade de Campinas (UNICAMP), 13083-863 Campinas, SP, Brazil; 2 Laboratório de Citogenética, Instituto de Ciências Biológicas, Universidade de Passo Fundo (UPF), CEP 99001-970 Passo Fundo, RS, Brazil; 3 Museu de Zoologia "Prof. Adão José Cardoso", Instituto de Biologia, Universidade Estadual de Campinas, 13083-863 Campinas, SP, Brazil; 4 Departamento de Biologia, Centro de Ciências da Saúde, Universidade Federal do Maranhão, 65085-580 São Luis, MA, Brazil; 5 Departamento de Ciências Básicas e Produção Animal, Faculdade de Agronomia, Medicina Veterinária e Zootecnia, Universidade Federal de Mato Grosso, 78060-900 Cuiabá, MT, Brazil; 6 Instituto Nacional de Pesquisas da Amazônia (INPA), 69060-001 Manaus, AM, Brazil; 7 Instituto de Pesquisas Científicas e Tecnológicas do Estado do Amapá, Divisão de Zoologia, 68912-250 Macapá, AP, Brazil

**Keywords:** *Phyllomedusa*, Morphological variation, Chromosome, Phylogenetic inference

## Abstract

**Background:**

The taxonomic and phylogenetic relationships of the genus *Phyllomedusa* have been amply discussed. The marked morphological similarities among some species hamper the reliable identification of specimens and may often lead to their incorrect taxonomic classification on the sole basis of morphological traits. Phenotypic variation was observed among populations assigned to either *P. azurea* or *P. hypochondrialis*. In order to evaluate whether the variation observed in populations assigned to *P. hypochondrialis* is related to that in genotypes, a cytogenetic analysis was combined with phylogenetic inferences based on mitochondrial and nuclear sequences.

**Results:**

The inter- and intra-population variation in the external morphology observed among the specimens analyzed in the present study do not reflect the phylogenetic relationships among populations. A monophyletic clade was recovered, grouping all the specimens identified as *P. hypochondrialis* and specimens assigned *P. azurea* from Minas Gerais state. This clade is characterized by conserved chromosomal morphology and a common C-banding pattern. Extensive variation in the nucleolar organizing region (NOR) was observed among populations, with four distinct NOR positions being recognized in the karyotypes. Intra-population polymorphism of the additional rDNA clusters observed in specimens from Barreiras, Bahia state, also highlights the marked genomic instability of the rDNA in the genome of this group. Based on the topology obtained in the phylogenetic analyses, the re-evaluation of the taxonomic status of the specimens from the southernmost population known in Brazil is recommended.

**Conclusions:**

The results of this study support the need for a thorough revision of the phenotypic features used to discriminate *P. azurea* and *P. hypochondrialis*. The phylogenetic data presented here also contribute to an extension of the geographic range of *P. hypochondrialis*, which is known to occur in the Amazon basin and neighboring areas of the Cerrado savanna, where it may be sympatric with *P. azurea*, within contact zones. The misidentification of specimens may have led to inconsistencies in the original definition of the geographic range of *P. azurea*. The variability observed in the NOR of *P. hypochondrialis* reinforces the conclusion that these sites represent hotspots of rearrangement. Intraspecific variation in the location of these sites is the result of constant rearrangements that are not detected by classical cytogenetic methods or are traits of an ancestral, polymorphic karyotype, which would not be phylogenetically informative for this group.

## Background

The taxonomic classification [1,2] and phylogenetic relationships [3,4] of the frogs of the genus *Phyllomedusa* have been subjected of extensive debate. Representatives of the genus are distributed throughout Central America and in South America east of the Andes, as far south as Argentina [2]. The genus *Phyllomedusa* is currently composed of 30 species, of which 26 have been allocated to four species groups, based on morphological features – the *P. burmeisteri* (5 spp.), *P. hypochondrialis* (9 spp.), *P. perinesos* (4 spp.), and *P. tarsius* (8 spp.) species groups [4,5]).

The marked morphological similarities of members of this genus hamper the reliable identification of species, often resulting in taxonomic inaccuracies, redefinition of species, and frequent description of new species. Based on morphological traits, Caramaschi [2] redefined the phenetic *P. hypochondrialis* species group, which currently consists of *P. azurea*, *P. centralis*, *P. hypochondrialis*, *P. megacephala*, *P. nordestina*, *P. oreades*, and *P. rohdei*[5]. Morphological data [6]) and phylogenetic inferences [4] indicate that *P. araguari*[7] is a synonym of *P. oreades*[6], while Baêta et al. [8] recognized *P. itacolomi*[9] as a synonym of *P. ayeaye*. These recent studies are indicatives of the taxonomic instability that the genus is still subjected.

A recent molecular phylogenetic analysis by Faivovich et al. [4] revealed the presence of two subclades within the *P. hypochondrialis* species group. One of these clades included *P. azurea*, *P. hypochondrialis*, and *P. nordestina*, while the other is composed of the remaining four species. There are numerous reports of taxonomic errors involving *P. azurea*, *P. hypochondrialis*, and *P. nordestina*, which have barely distinguishable diagnostic characteristics [4]. *Phyllomedusa azurea* is known to occur in open habitats of the Cerrado savannas, Pantanal wetlands, and Chaco scrub biomes, whereas *P. hypochondrialis* is distributed mainly in the Amazonian region and areas of Amazonian influence in the Pantanal [4] and *P. nordestina* is found in wet habitats amidst the Caatinga scrublands of the Brazilian Northeast. Recent records of *P. azurea* extend previously known distribution to a Cerrado-Amazon transitional zone in the state of Rondônia [10], and to open upland habitats in Santa Catarina, southern Brazil [11]. However, the correct taxonomic classification of these populations is still unclear [11].

Chromosomal characteristics of members of the genus *Phyllomedusa* are relatively poorly known, although the karyotypes of a number of species have been described, including *P. rohdei*[12-14], *P. camba*[12], *P. nordestina*[14], *P. hypochondrialis*[15], *P. distincta*[16], and *P. tetraploidea*[16-18]. However, potentially informative chromosomal features have been observed, in particular, the marked variability in the number and position of the NORs in different populations [12,13,15,16]. Morphological variation has also been observed in some populations assigned to *P. hypochondrialis*[4] suggesting the need for the complementary application of different interpretative tools as a helpt to clear their taxonomic status.

Considering these fundamental problems, the present study focused on the morphological variation found in frog populations attributed to *P. hypochondrialis*, and examined whether this variation is interspecific or inter-populational. Cytogenetic and molecular approaches are also used in order to verify whether the observed phenotypic variation is related to genotype-level variation, based on the analysis of specimens obtained from Brazilian populations assigned to *P. hypochondrialis* and *P. azurea* from a number of distinct regions. It was also considered specimens that could not be safely identified up to the specific level, such as *Phyllomedusa* cf. *hypochondrialis*, *Phyllomedusa* sp. (aff. *hypochondrialis*).

## Results

### Morphological analysis

**Figure 1:**
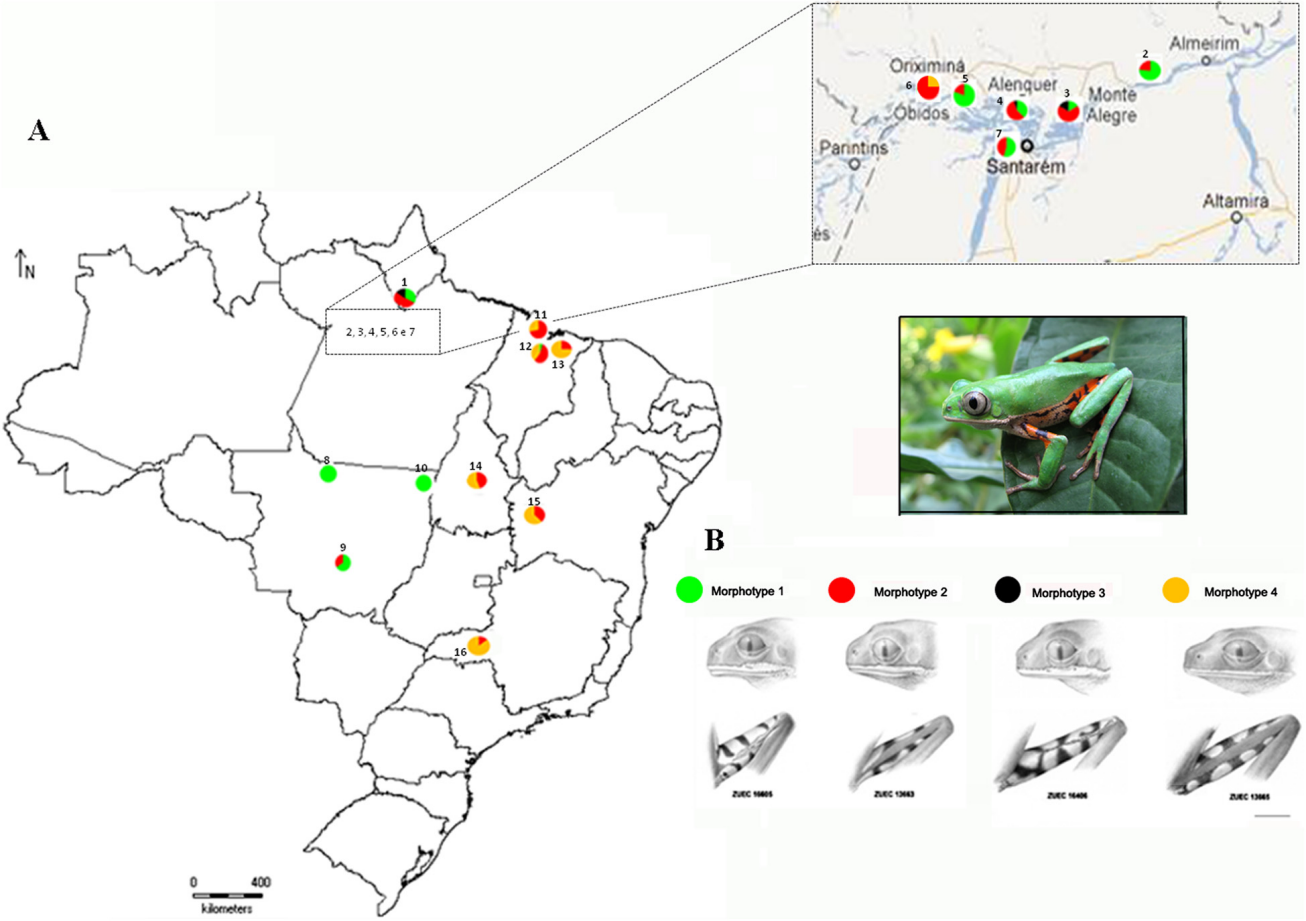
**Distribution of the morphological variation in populations assigned*Phyllomedusa hypochondrialis*. (A)** Geographic location of the sites in Brazil at which specimens were collected for the present study. The circles represent the percentage occurrence of the four morphotypes in each population. **(B)** Representative morphotypes 1 (green), 2 (red), 3 (black) and 4 (yellow).

The variation in external morphology observed among the specimens allowed the recognition of four morphotypes, based on the diagnostic traits used to distinguish *P. azurea* from *P. hypochondrialis* (Figure 1):

Morphotype 1 Narrow white stripe on the upper lip extending to the lower eyelid together with the presence of a discontinuous, wide green stripe along 2/3 to 3/4 of the length of the upper surface of the thighs. Specimens presenting these character conditions correspond to *P. hypochondrialis*, according to Caramaschi [2].

Morphotype 2 White stripe on the upper lip extending to the lower eyelid together with the presence of a wide green stripe along the full length of the upper surface of the thighs.

Morphotype 3 White stripe on the upper lip extending to the lower eyelid. Green stripe absent on the upper surface of the thighs.

Morphotype 4 White stripe on the upper lip that does not extend as far the lower eyelid. Wide green stripe along the full length of the upper surface of the thighs. Specimens presenting these character conditions correspond to *P. azurea*, according to Caramaschi [2].

Two of the other morphological characteristics used by Caramaschi [2] to distinguish *P. azurea* and *P. hypochondrialis* vary considerably both within and between populations, and cannot be used reliably to identify the species: the size of the adhesive discs relative to the eardrum (discs larger than the eardrum in *P. hypochondrialis* and smaller in *P. azurea*) and the white stripe on the upper lip, which is visible dorsally in *P. hypochondrialis*, but not in *P. azurea*.

In the present study, the four morphotypes were distributed among the different populations, and with the exception of Alta Floresta (population L8) and Santa Terezinha (L10), which were each represented by a single specimen, all the populations presented at least two distinct morphotypes (Figure 1 and Table 1). While there was no clear geographic pattern, specimens classified as morphotypes 1 and 2 predominated in populations from the Brazilian states of Amapá, Pará, and Mato Grosso, while morphotypes 2 and 4 were prevalent in populations from Maranhão, Tocantins, Bahia, and Minas Gerais.

**Table 1:** Distribution of the four recognized morphotypes in each study population

	**Population**			**Morphotype**		
		**N**	**1**	**2**	**3**	**4**
**L1-**	Laranjal do Jari/AP	21	34%	52%	14%	*
**L2-**	Prainha/PA	22	77%	23%	*	*
**L3-**	Monte Alegre/PA	6	16%	68%	16%	*
**L4-**	Alenquer/PA	16	38%	57%	5%	*
**L5-**	Oriximiná/PA	5	80%	20%	*	*
**L6-**	Óbidos/PA	7	*	75%	*	25%
**L7-**	Belterra/PA	6	55%	45%	*	*
**L8-**	Alta Floresta/MT	*1*	*100%*	*	*	*
**L9-**	Chapada dos Guimarães/MT	6	66%	34%	*	*
**L10-**	Santa Terezinha/MT	*1*	*100%*	*	*	*
**L11-**	São Luís/MA	11	*	72%	*	28%
**L12-**	Bacabeira/MA	18	5%	56%	*	39%
**L13-**	Urbano Santos/MA	8	*	25%	*	75%
**L14-**	Porto Nacional/TO	13	*	38%	*	62%
**L15-**	Barreiras/BA	18	*	45%	*	55%
**L16-**	Uberlândia/MG	7	*	14%	*	86%

Asterisks (*) indicate the absence of the respective morphotype.

### Phylogenetic inferences

**Figure 2:**
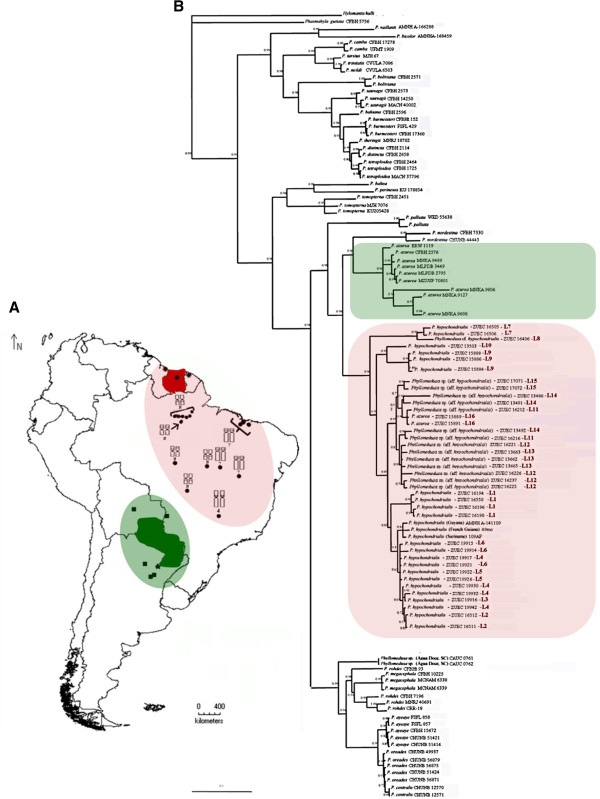
**Geographical distribution of the sequences and topology of*Phyllomedusa*produced by Bayesian analysis: (A)** Topographic map of South America: squares represent the *P. azurea* sequences obtained from GenBank, stars represent karyotypes described by Morand and Hernand (1997); Paraguay (green) is the type locality of *P. azurea* and green shaded region represent the distribution of haplotypes in the same clade; asterisks represent *P. hypochondrialis* sequences from GenBank; Suriname (red) is the type locality of *P. hypochondrialis*, the red shaded region represents the distribution of *P. hypochondrialis* haplotypes in the same clade. Note the distribution of NOR positions in the red clade in the populations samples. **(B)** consensus Bayesian Inference (BI) of the 2928-bp concatenated sequence of mitochondrial (12S and 16S) and nuclear (Rag-1) genes in *Phyllomedusa* based on the GTR+R+I model. Bayesian posterior probabilities values are shown at each node. Nodes with support of 1.0 are marked with asterisks.

The Bayesian inference recognized the four *Phyllomedusa* species groups – *P. burmeisteri*, *P. tarsius*, *P. perinesos*, and *P. hypochondrialis* groups – as monophyletic (Figure 2), as reported by previous authors. In the specific case of the *P. hypochondrialis* group, two distinct and well-supported subclades were also identified. One of these subclades includes *P. azurea*, *P. hypochondrialis*, and *P. nordestina*, while the other contains *P. rohdei, P. megacephala*, *P. centralis*, *P. araguari*, and *P. oreades*.

All the populations tentatively assigned to *P. hypochondrialis* [*P. hypochondrialis, Phyllomedusa* sp. (aff. *hypochondrialis), Phyllomedusa* cf. *hypochondrialis*)] formed a monophyletic clade together with the GenBank sequences of specimens from the Guyanas (96% posterior probability for Bayesian Inference-Figure 2). By contrast, specimens tentatively attributed to *P. azurea* from Uberlândia (L16), state of Minas Gerais – in the Cerrado biome– were paraph yletic in relation to the *P. azurea* haplotypes from Argentina, Bolivia, and Paraguay. In fact, the specimens from Uberlândia grouped with the *P. hypochondrialis* clade.

In the BI topology, the specimen from Alta Floresta, Mato Grosso (L8), formed a subclade with the population from Belterra, in Pará (L7), with aposterior probability of 99%. A second subclade was composed of populations from Chapada dos Guimarães and Santa Teresinha, both in Mato Grosso (posterior probability 99%). The third subclade included haplotypes from Maranhão (L11-L13), Tocantins (L14), Bahia (L15) and Minas Gerais (L16), with a posterior probability of 95%. Finally, the specimens from Amapá (L1) and some localities in the state of Pará (L2-L6) grouped with the GenBank sequences of one specimen from Suriname (the type locality of *P. hypochondrialis*) and two from French Guiana and Guyana.

These results also provide useful insights into the taxonomic status of a population recently discovered in Água Doce, in the Brazilian state of Santa Catarina by Lucas et al. [11], which is morphologically similar to *P. azurea*. However, the haplotypes of the specimens from this population were paraphyletic in relation to the other haplotypes of the *P. azurea* clade, and were closely related to the second major clade in the *P. hypochondrialis* group (*P. rohdei*, *P. ayeaye*, *P. centralis*, *P. megacephala* and *P. oreades*) inferred in phylogenetic reconstruction.

### Cytogenetic data

#### Description of the karyotypes

**Figure 3:**
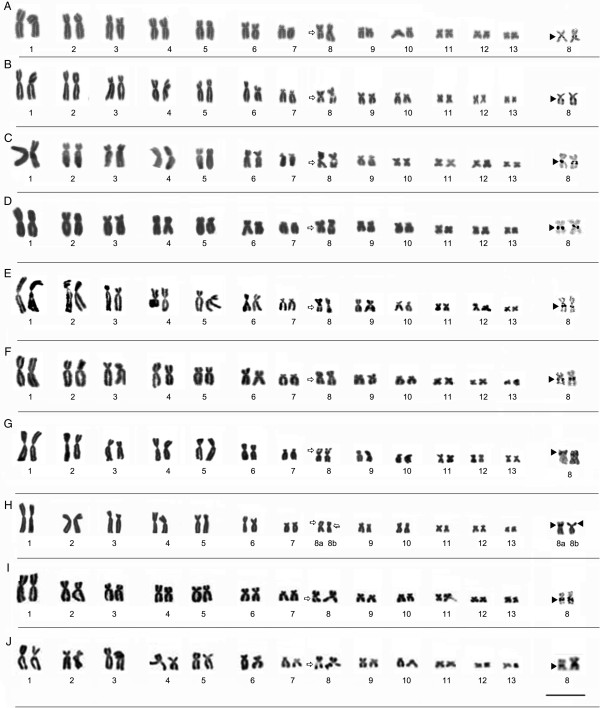
**Giemsa-stained karyotypes and chromosome pairs sequentially submitted the Ag-NOR. (A)** L1- Laranjal do Jari/AP (ZUEC 19196, male), **(B)** L2- Prainha/PA (ZUEC 16512, male), **(C)** L3- Monte Alegre/PA (ZUEC 19916, male), **(D)** L4- Alenquer/PA (ZUEC 19932, female), **(E)** L5- Óbidos/PA (ZUEC 19914, male), **(F)** L6- Oriximiná/PA (ZUEC 19922, male), **(G)** L7- Belterra/PA (ZUEC 16506, male), **(H)** L8- Alta Floresta/MT (ZUEC 16406, male), **(I)** L9- Chapada dos Guimarães/MT (ZUEC 15886, male), and **(J)** L10- Santa Terezinha/MT (ZUEC 13503, male). The arrows indicate secondary constrictions. Insets (right) show NOR-bearing chromosomes. Chromosomes were sequentially impregnated by silver nitrate after standard staining in **C**-**G**. The arrowheads indicate NOR sites. Note the heteromorphic position of the NOR site in the karyotype from Alta/Floresta **(H)**: pericentromeric in morph 8a and subterminal in morph 8b. Bar=3 μm.

**Figure 4:**
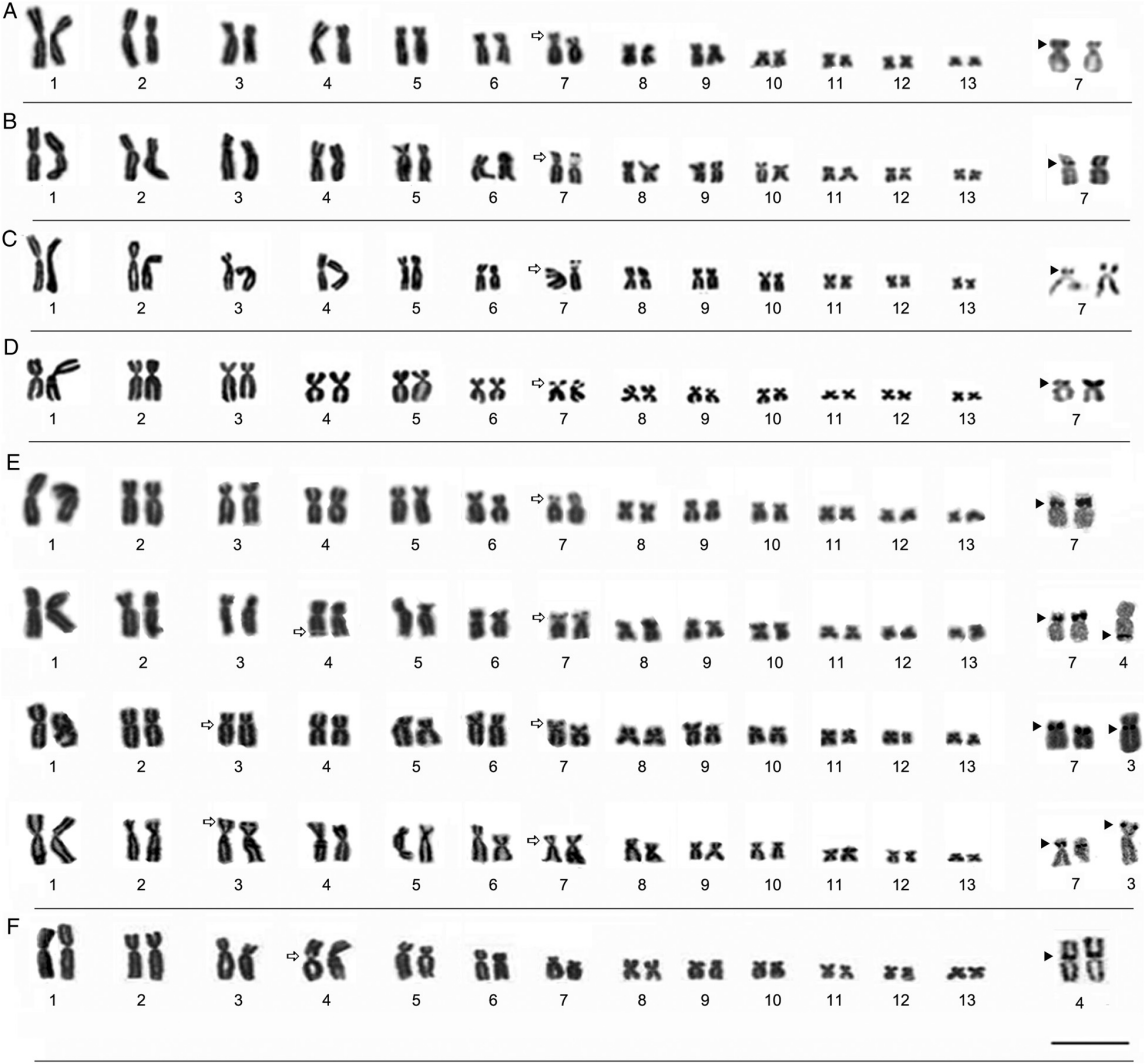
**Giemsa-stained karyotypes and chromosome pairs sequentially submitted the Ag-NOR method. (A)** L11- São Luís/MA (ZUEC 16216, male), **(B)** L12- Bacabeira/MA (ZUEC 16225, male), **(C)** L13- Urbano Santos/MA (ZUEC 13665, female), **(D)** L14-Porto Nacional/TO (ZUEC 13491, male), **(E)** L15- Barreiras/BA with four different specimens (ZUEC 17075, male; ZUEC 17072, male; ZUEC 17071, male and ZUEC 17082, male) showing additional secondary constrictions and NOR sites and **(F)** L16-Uberlandia/MG (ZUEC 15888, male). The arrows indicate the secondary constrictions. Insets (right) show NOR-bearing chromosomes and arrowheads indicate NOR sites. Bar=3 μm.

All the specimens analyzed presented a diploid number of 2n = 26 chromosomes (Figures 3 and 4). The karyotype consisted of four metacentric (1, 4, 8, 11), six submetacentric pairs (2, 3, 5, 6, 12, and 13) and three subtelocentric (7, 9 and 10). Pair 7 was variable among populations, it was characterized as subtelocentric in the specimens from Amapá (L1), Pará (L2-L7), and Mato Grosso (L8-L10) and submetacentric in the specimens from Maranhão (L11-L13), Tocantins (L 14), Bahia (L15) and Minas Gerais (L16).

### Nucleolar organization region (NOR)

**Figure 5:**
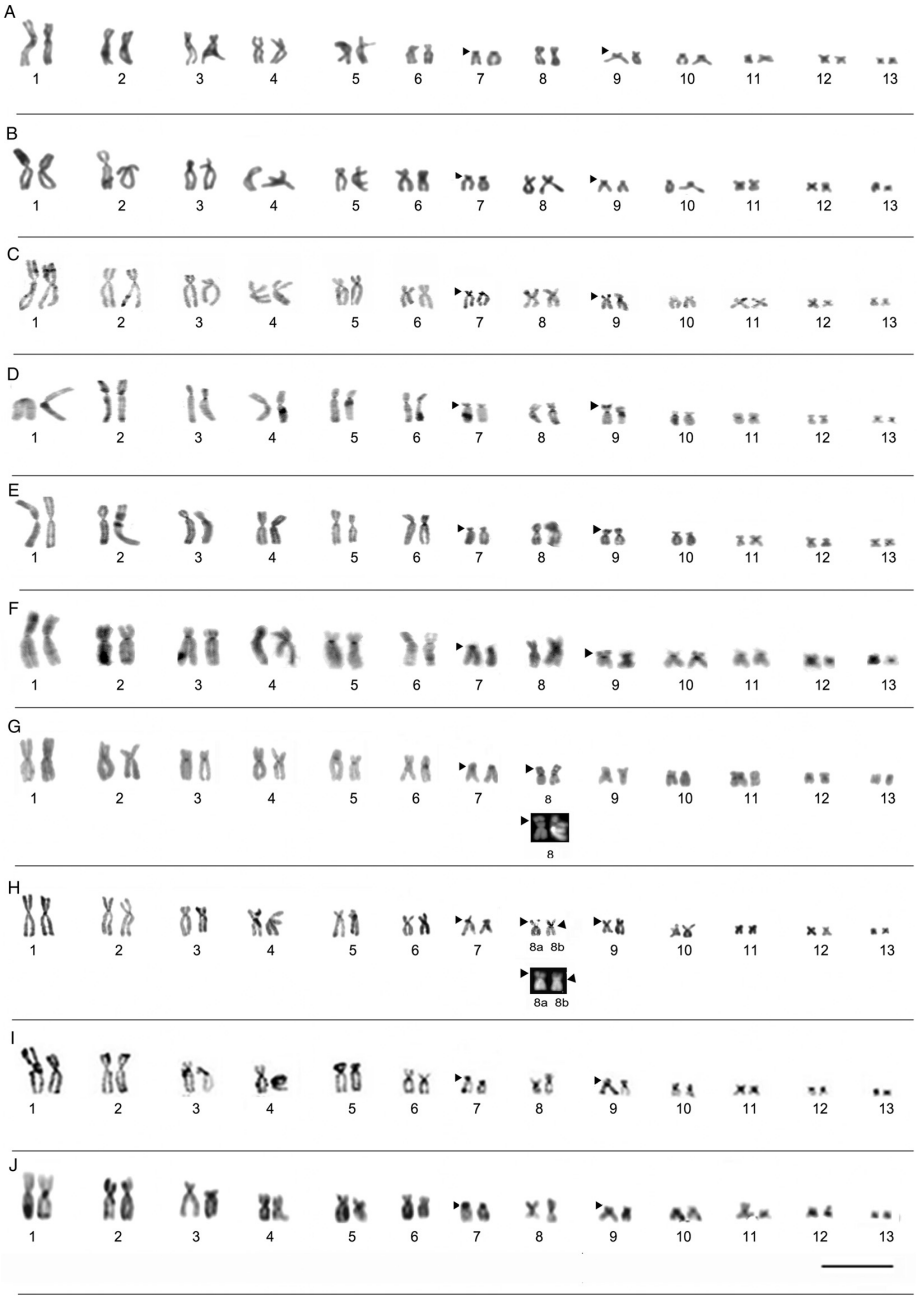
**Karyotypes defined by C-banding. (A)** L1- Laranjal do Jari/AP (ZUEC 19196, male), **(B)** L2- Prainha/PA (ZUEC 16512, male), **(C)** L3- Monte Alegre/PA (ZUEC 19916, male), **(D)** L4- Alenquer/PA (ZUEC 19932, female), **(E)** L5- Óbidos/PA (ZUEC 19914, male), **(F)** L6- Oriximiná/PA (ZUEC 19922, male), **(G)** L7- Belterra/PA (ZUEC 16506, male), **(H)** L8- Alta Floresta/MT (ZUEC 16406, male), **(I)** L9- Chapada dos Guimarães/MT (ZUEC 15886, male), and **(J)** L10- Santa Terezinha/MT (ZUEC 13503, male). The arrowheads indicate the non-centromeric heterochromatic blocks. Note that in **(G)** and **(H)**, pair 8 was stained using the C-banding technique, and the same pair was sequentially stained using DAPI (below). The negatively stained region coincided with the secondary constriction, which was homomorphic in the karyotype from Belterra **(G)** and heteromorphic in that from Alta Floresta **(H)**, thus confirming inversion in this region. Scale Bar=3 μm.

The location of NORs varied among populations. In the karyotype of populations from Amapá, Pará, and Mato Grosso, NORs were observed inthree different positions on chromosome 8. In other Amazonian populations, from Amapá (L1), Pará (L2, L3, L4, L5, and L6), and Mato Grosso (L9 and L10), NORs were detected in the pericentromeric region of the long arm, coinciding with secondary constrictions revealed by the Giemsa staining (Figure 3). In the Belterra specimens (Figure 3G), secondary constrictions and DAPI staining were observed in the subterminal region of the short arm of chromosome 8, corresponding to the position of the NOR identified by the Ag-NOR method. In the single specimen from Alta Floresta (L8), heterozygous NOR was observed in pair 8. In morph 8a, the NOR was located in the pericentromeric region while in morph 8b, it was found in the subterminal region (Figure 3H). The heteromorphic condition was also confirmed by Giemsa, C-banding, DAPI, and Ag-NOR staining, and no difference in the arm ratio or chromosome size was observed (Figures 3 and 5).

In specimens from Maranhão (L11, L12 and L13, respectively, from São Luí s, Bacabeira, Urbano Santos) and from Porto Nacional, in Tocantins (L14), NORs were located in the interstitial region of the short arm of pair 7, coinciding with the secondary constrictions observed by Giemsa staining (Figure 4). Intra-population variation was also observed in specimens from Barreiras (L15), in which the NOR was located in the interstitial region of the short arm of pair 7 in all individuals. Additional NOR was observed in subterminal region of one homologue of pair 4 (specimen ZUEC 17072), in the subterminal region of the short arm in one homologue of pair 3 (specimens ZUEC 17082 and 17083), and in the pericentromeric region of the long arm of one homologue of pair 3 (specimens ZUEC 17071 and 17078) (Figure 4E). In all these cases, the regions were identified as secondary constrictions by Giemsa staining. In specimens from Uberlândia (L16), NORs were detected in the pericentromeric region of the short arm of pair 4, coinciding with secondary constrictions (Figure 4F).

#### Heterochromatic patterns

**Figure 6:**
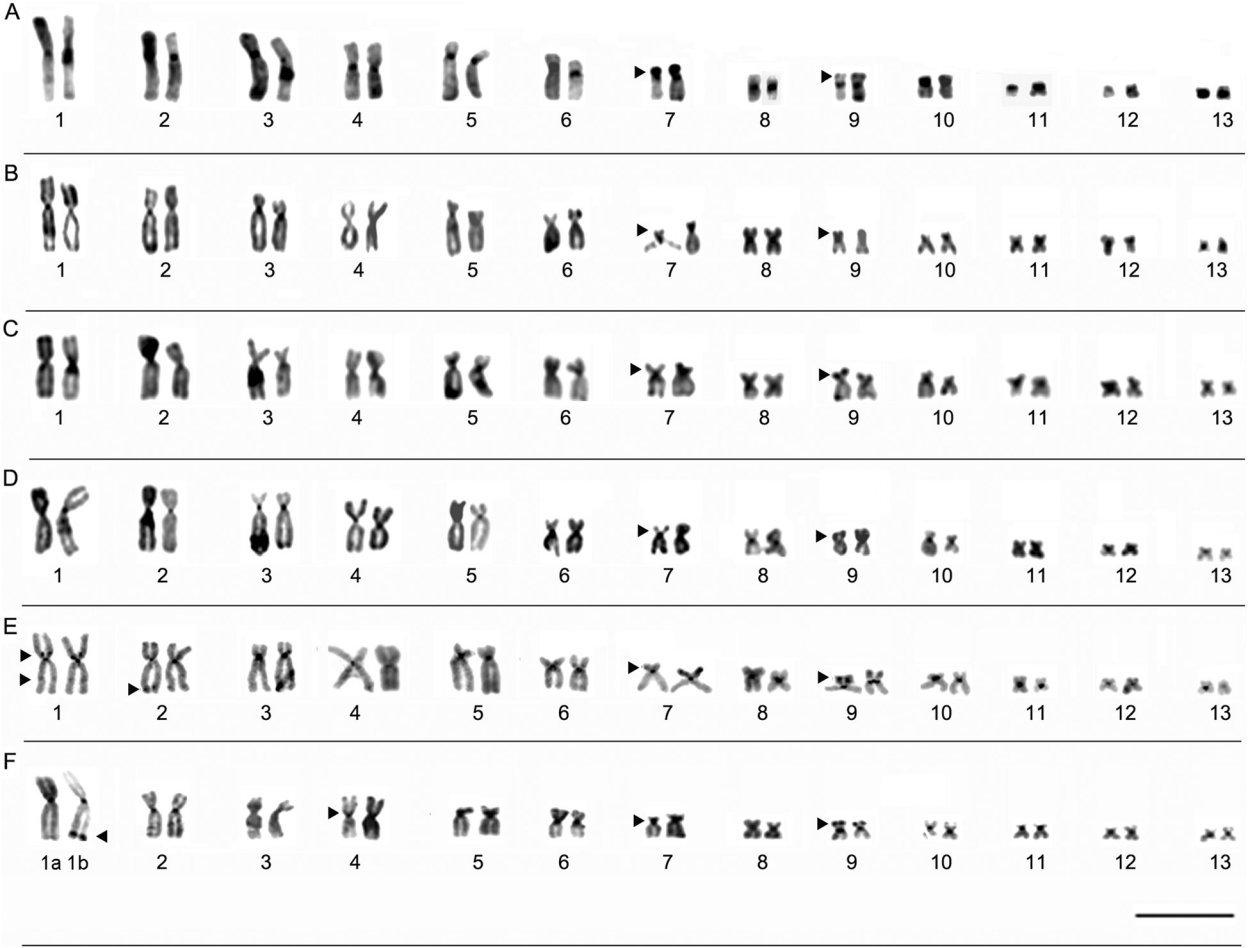
**Karyotypes defined by C-banding. (A)** L11- São Luís (ZUEC 16216, male), **(B)** L12- Bacabeira (ZUEC 16225, male), **(C)** L13- Urbano Santos (ZUEC 13665, female), **(D)** L14- Porto Nacional (ZUEC 13491, male), **(E)** L15- Barreiras (ZUEC 17072, male), and **(F)** L16- Uberlândia (ZUEC 15888, male). The arrowheads indicate th e position of the non-centromeric heterochromatic blocks. Note in **(F)** subterminal block of heterochomatin in a single homologue of pair 1 (morph 1b), absent in morph 1a. Bar=3 μm.

The C-banding technique revealed heterochromatic blocks in the centromeric regions of all the chromosomes examined. Pericentromeric blocks were detected on the short arms of pair 7 in all specimens, extending from the pericentromeric to the subterminal region (Figures 5 and 6). All the karyotypes also presented a subterminal heterochromatic block in the short arm of submetacentric pair 9 (Figures 5 and 6). Heteromorphisms were observed in the short arm of pair 8 from the Alta Floresta population by staining Giemsa and Ag-NOR methods (Figure 3H). These pair, when submitted to C-banding showed inversion in heterochromatin block detected in this arm: in the morph 8a, the heterochromatin was detected in subterminal position and in 8b morph the C-positive block was detected in the pericentromeric position (Figure 5H). The C-banded slides were sequentially stained with DAPI and showed DAPI-positive band in the same position of the heterochromatin block (Figure 5H) and clearing confirmed the inversion in this chromosomal region. In the Belterra specimens (Figure 5G), C-positive block and DAPI-positive band were observed in the subterminal region of the short arm of chromosome 8.

Additionally, C-bands were observed in the pericentromeric region of the short arm and interstitially on the long arm of pair 1 (Figure 6E) and in the subterminal region of the long arm of pair 2 in specimens from Barreiras (L15). A pericentromeric block was also observed in the short arm of pair 4 and a subterminal block of heterochomatin in a single homologue of pair 1 (morph 1b) in specimens from Uberlândia (L16), not detected in morpho 1a (Figure 6F). Morph 1b was observed in all the metaphases analyzed, irrespective of the sex of the specimen.

## Discussion

The phylogenetic analyses of the genus *Phyllomedusa* presented here support emphatically the monophyletic status of the *P. burmeisteri*, *P. tarsius*, *P. perinesos* and *P. hypochondrialis* species groups, further reinforcing the topology obtained by Faivovich et al. [4] However, a number of questions remain with regard to the group-level classification of certain species. The analyses cluster all the Brazilian specimens examined (L1-16) in the same clade, together with sequences from Suriname, French Guiana, and Guyana. The Brazilian populations (L1-16) analyzed here were all identified as *P. hypochondrialis*, based on the fact that the type locality of *P. hypochondrialis* was identified as “Suriname” in the original description (see reference [5]). The inter- and intra-population variation in external morphology observed among the specimens of this clade does not reflect the differences in the phylogenetic relationships among populations.

Morphotype 1 corresponds to the set of characteristics used to describe the species *P. hypochondrialis*, while morphotype 4 corresponds to the description of *P. azurea*. Morphotype 2 corresponds to a mixture of the diagnostic traits of the two species, while morphotype 3 combines characteristics that do not correspond to any formal species description. Overall, then, the results of the present study indicate that diagnostic traits employed by Caramaschi [2] for the differentiation of *P. hypochondrialis* and *P. azurea* are in fact combined in varying proportions in the populations examined. In this case, it is not possible to identify any specific morphological pattern associated with the geographic distribution of the populations. The high frequency of intermediate morphotypes in individuals of these two species (morphotype 2) re-emphasizes the difficulties in morphologically distinguishing *P. azurea* from *P. hypochondrialis*. The morphological variation observed in the present study supports the need for a careful re-analysis of the phenotypic features used to discriminate *P. azurea* and *P. hypochondrialis*.

Chromosomal morphology and C-banding patterns are conserved within the *P. hypochondrialis* clade, indicating the presence of homologies among the different karyotypes. While there is some variation in the morphology of pair 7 (ST/SM) in population L11-15 in comparison with the other populations (L1-10, L16), it is possible to infer homologies between these karyotypes, which can be explained by the presence of NORs in submetacentric pair 7, the increase in the arm ratio, and the centromeric position of this pair. Other common features in this clade include the distribution of heterochromatin in pairs 7 (pericentromeric) and 9 (subterminal), which appears to be a diagnostic feature of *P. hypochondrialis*.

The topology obtained from the phylogenetic analysis indicated the presence of subclades within *P. hypochondrialis*, related to variations in the position of the rDNA cluster in the genome. The subclade formed by specimens from Belterra (L7) and Alta Floresta (L8) is related to the presence of NORs in the short arm of pair 8. Despite the presence of a paracentric inversion involving NOR segments in pair 8 (morph 8b) in the single specimen from Alta Floresta (L8), the 8a morph in this karyotype appears to be homologous with pair 8 in the specimens from Belterra (L9). The NOR position detected by the Ag-NOR method and the heterochromatic block detected by C-banding and DAPI staining support this conclusion. This type of rearrangement has been reported in other anurans, such as *Agalychnis*[19] and *Scythrophrys*[20]. Nevertheless, examination of additional specimens from the populations analyzed here would be necessary for a more conclusive understanding of NOR dynamics in these animals.

Two *P. hypochondrialis* subclades presented NORs in a pericentromeric position on long arm of the pair 8. One subclade included specimens from Mato Grosso – Chapada dos Guimarães (L10) and Santa Terezinha (L9) – while the ot her encompassed the populations from Amapá (L1) and Pará (L2-L6), together with Suriname, French Guiana, and Guyana. Despite this similarity, the shared trait is not phylogenetically informative for the diagnosis of the two groups.

The subclade composed of populations from Maranhão (L11-L13), Tocantins (L14), Bahia (L15), and Minas Gerais (L16) presents a complex and potentially interesting pattern of NOR variation. Within this subclade, some populations had NOR fixed in pair 7 (L11-L14), while others had NOR in pair 4 (L16). In the population from Bahia (L15), NOR was observed primarily in pair 7 (all specimens), but there was also a polymorphism in the additional rDNA clusters, which were distributed in distinct patterns in different specimens. Additional NOR has been recorded in other anurans (e.g., [19,21-23]).

The distribution (number and position) of rDNA clusters in the genome has been used as a chromosomal marker in cytogenetic studies of a range of taxonomic groups, and in some cases, it has been useful for the discrimination of species [21,24,25] and the interpretation of phylogenic relationships [26-28]. However, the usefulness of this characteristic for the diagnosis of phylogenetic relationships must be assessed carefully, given that the variation in the location of NORs is not necessarily a reliable indicator of the distinction between taxa, and in some cases must be interpreted as variation between populations.

The extensive NOR variation within species and between populations observed in the present study impedes a reliable interpretation of the evolution of this characteristic in the study group. A similar pattern of NOR variation has been observed in other *Phyllomedusa* species, such as *P. rohdei*[12,13], *P. camba*[12], *P. ayeaye* (Bruschi - personal observation; [29]), *P. burmeisteri*[29], *P. tarsius*[29], *P. tetraploidea*[16,29], *P. distincta*[16,29]. This suggests recurrent variation in this characteristic during the course of the evolutionary history of this group. The recurrent variation in the position of the NORs may reflect either the rapid rate of evolution of this character in this genus or a polymorphic ancestral karyotype.

Studies of a number of different taxonomic groups indicate that NOR sites represent unstable regions of the genome and therefore are important “hotspots” of rearrangement [30-36]. These clusters have a number of features in common with other rearrangement hotspot regions, such as the presence of tandem repeats [36], and play an important role in non-homologous recombination [37]. The association of a transposon with clusters of rDNA is thought to contribute to the instability of the genome in these regions [38,39].

The results of the present study reconfirm Caramaschi’s [2] revalidation of *P. azurea* as a *taxon* distinct from *P. hypochondrialis*, and reinforce the conclusions of Faivovich et al. [4]. These results also confirm the need for a more precise morphological definition of each species, given that some *P. hypochondrialis* populations presented characteristics thought to be diagnostic of *P. azurea.* This was case of specimens from Uberlândia, Minas Gerais state, tentat ively attributed to *P. azurea*- in the Cerrado biome, where the species typically occurs [2,40]) – but in Bayesian inference was recovered in *P. hypochondrialis* clade, suggesting a misidentification. The inclusion of the population from Água Doce (Santa Catarina) - identified by Lucas et al. (2011) as *P. azurea* – in ours analysis reinforced the difficulties of differentiating these taxa based on external morphology. In fact, the morphological variation observed in the specimens from Água Doce indica tes the possible presence of a species complex in *P. azurea*. In the present study, this population was included in the second major clade of the *P. hypochondrialis* group, and was paraphyletic in relation to the *P. azurea* haplotypes from Paraguay, Bolivia, and Argentina. The topology obtained here indicates that the identification of the Água.

Doce population must be reevaluated, preferably through the integrated analysis of chromosomal, morphological, and bioacoustic data. Interestingly, Água Doce is located within the Pampas biome, which is characterized by extensive grassland habitats [41], also characteristic of the ecosystems inhabited by other species included in the second subclade of the *P. hypochondrialis* group, such as *P. megacephala*, *P. ayeaye*, *P. centralis*, and *P. oreades*, which are also found in plateau and upland areas. The only exception is *P. rohdei*, found in the Brazilian Atlantic Forest.

## Conclusion

The *P. hypochondrialis* samples analyzed in the present study confirmed the considerable morphological variation found among populations, and reinforced the need for more systematic phenotypic studies for the definition of reliable diagnostic features for the identification of *P. hypochondrialis* and *P. azurea*. The results of the present phylogenetic analysis also contributed to the extension of the known geographic distribution of *P. hypochondrialis*, previously known only from the Amazon region, to areas of open Cerrado savanna (such as Uberlândia/Minas Gerais State), including areas of possible sympatry with *P. azurea*. Additionally the misidentification of *P. azurea* may have led to mistakes in this species’ range limits.

The analysis of chromosomal markers allowed the identification of homologies and contributed to a better understanding of chromosomal evolution in this genus. Interestingly, the observed NOR variability in *P. hypochondrialis* reinforces the suggestion that NOR sites are hotspots of rearrangement and that the intraspecific variation in the location of these sites is either the result of processes that are not detected by classical cytogenetic methods or the remnants of a polymorphic ancestral karyotype.

## Methods

### Biological samples

A total of 166 specimens of *Phyllomedusa* were collected from sixteen Brazilian sites (L1 to L16: Figure 1 and Table 2). The collection of specimens was authorized by the Brazilian federal environment institute (IBAMA – license 20266–1). Voucher specimens were deposited in the Museu de Zoologia “Prof. Dr. Adão José Cardoso” (ZUEC), at Universidade Estadual de Campinas (UNICAMP) in São Paulo state, Brazil. The complete list of voucher numbers, GenBank accession numbers, and collecting localities are provided in Table 2.

**Table 2:** Species, code in the map, collecting localities of the samples examined in morphological, phylogenetic and cytogenetic analysis

**Species**	**Code**	**Locality/State**		**Specimens**	
			**Morphological**	**Molecular**	**Cytogenetic**
*P. hypochondrialis*	L1	Laranjal do Jari/AP	16194-16199; 16550-16564	16194; 16196; 16198; 16550	16194-16199
*P. hypochondrialis*	L2	Prainha/PA	16510-16532	16511; 16512	16510-16515
*P. hypochondrialis*	L3	Monte Alegre/PA	19916; 19929; 19938; 19940; 19944;	19916	19916; 19929; 19938;
			19945		19940; 19944; 19945
*P. hypochondrialis*	L4	Alenquer/PA	19917; 19920; 19923–19925; 19927;	19917; 19930; 19932; 19942	19917; 19920; 19923–19925;
			19930; 19932; 19935–19937; 19941;		19927; 19930; 19932; 19935-
			19942; 19946-19948		19937
*P. hypochondrialis*	L5	Oriximiná/PA	19918, 19922; 19926; 19928; 19929	19922; 19926	19918, 19922; 19926;
					19928; 19929
*P. hypochondrialis*	L6	Óbidos/PA	19914; 19945; 19919; 19921; 19933	19914; 19915; 19921	19914; 19945; 19919;
			19934; 19943		19921; 19933
*P. hypochondrialis*	L7	Belterra/PA	16504-16509	16505; 16506	16504-16509
*Phyllomedusa* cf. *hypochondrialis*	L8	Alta Floresta/MT	16406	16406	16406
*Phyllomedusa* cf*. hypochondrialis*	L9	Chapada dos Guimarães/MT	15883-15888	15884; 158 86;	15883-15888
				15888	
*Phyllomedusa* cf. *hypochondrialis*	L10	Santa Terezinha/MT	13503	13503	13503
*Phyllmedusa* sp. (aff. *hypochondrialis*)	L11	São Luís/MA	16208-16218	16212; 16216	16208; 16209; 16211;
					16215; 16217
*Phyllomedusa* sp. (aff. *hypochondrialis*)	L12	Bacabeira/MA	16221-16238	16221; 16225; 16226; 16237;	16224; 16228; 16229;
					16223; 16235-16238
*Phyllomedusa* sp. (aff.*hypochondrialis*)	L13	Urbano Santos/MA	13662-13669	13662; 13663; 13665	13662-13669
*Phyllomedusa* sp. (aff. *hypochondrialis*)	L14	Porto Nacional/TO	13486-13495; 13506-13508	13486; 13491; 13492	13486 - 13495; 13506-13508
*Phyllomedusa* sp. (aff. *hypochondrialis*)	L15	Barreiras/BA	17071-17088	17071; 17072	17071-17088
P. azurea*	L16	Uberlândia/MG	15882; 15889-15894	15889; 15891	15882; 15889–15894; 13474

* The nomenclature *P. azurea* correspond to prior identification this population before the analysis.

### Morphological variation

The two principal morphological characters identified by Caramaschi [2] for the diagnosis of *P. azurea* and *P. hypochondrialis* were evaluated in the present study: (1) the presence of a narrow white stripe on the upper lip and (2) the presence/absence and configuration of the green stripe on the upper surface of the thighs. All specimens collected were analyzed and photographed in a Zeiss stereomicroscope. Cytogenetic and molecular analyses of all specimens were conducted to assess the reliability of the morphology-based diagnosis.

### Isolation, amplification, and sequencing of DNA

Genomic DNA was extracted from liver or muscle tissues and stored at −70°C in the tissue bank at the Department of Structural and Structural Biology of the Campinas State University (Unicamp) in Campinas, São Paulo, Brazil, using t he TNES method as applied by Bruschi et al. [14]. The mitochondrial tRNA-Val, 12S and 16S ribosomal genes were amplified using the primers MVZ 59 (L), MVZ 50 (H), 12L13, Titus I (H), Hedges16L2a, Hedges16H10, 16Sar-L and 16Sbr-H (for primer sequences, see reference [42]). The nuclear gene RAG-1 was amplified using the primers RAG-1R and RAG-1F [3]. The amplified PCR products were purified with a GFX PCR and Gel Band DNA Purification kit (GE Healthcare, England) and used directly as templates for sequencing in an automatic ABI/Prism DNA sequencer (Applied Biosystems, Foster City, CA, USA) using the BigDye Terminator kit (Applyed Biosystems, Foster City, CA, USA), as recommended by the manufacturer. The DNA sequences were sequenced bi-directionally, edited in Bioedit version 7.0.1 (http://www.mbio.ncsu.edu/BioEdit/ bioedit.html), and aligned using Clustal W.

### Analysis of molecular data

The phylogenetic relationships among the populations were inferred from the concatenated matrix of the mitochondrial DNA 12S, tRNAval, and 16S rDNA sequences and nuclear Rag-1 gene (totalized 2920pb). To evaluate this approach, we selected 39 specimens analyzed by morphology and cytogenetic methods, representing at least one specimens to each morphotype observed within population. The data matrix was complemented with 70 *Phyllomedusa* sequences available in GenBank (Additional file 1). The species used as outgroups were *Hylomantis hulli* and *Phasmahyla guttata*, which were chosen based on the topology reported by Faivovich et al. [4]. The sequence was aligned using the Clustal W program [43]. The initial alignments were checked by eye and adjusted wherever necessary. Phylogenetic trees were constructed using Bayesian inference, based on the Markov chain Monte Carlo (MCMC) analysis, in MrBayes 3.1.2 [44] with two independent runs, each with four chains and sampling every 1000 generations for 6 million generations. The adequate burn-in (the first 25% trees excluded) was determined by examining a plot of the likelihood scores of the heated chain for convergence and stationary. The most appropriate evolutionary model selected by Modeltest 3.7 for the BI analysis was the GTR+R+I model [45].

### Cytogenetic analysis

Based in morphotype variation, we selected 110 specimens (complete description to specimens used are describes in Table 2) to submitted cytogenetic methods, representing each one of the morphotypes found in the screened populations. Metaphase cells were obtained from intestines and testes of animals previously treated with 2% colchicine, following procedures modified from King and Rofe [46] and Schmid [47]. Prior to the removal of the intestine and testes, the animals were anesthetized profoundly. Cell suspensions were dripped onto clean plates and stored at −20°C. The chromosomes were stained with 10% Giemsa, silver stained by the Ag–NOR method [48], and C-banded [49]. In two populations (L7 and L8), the C-banded chromosomes were also stained with DAPI (500 μg/mL), after Giemsa distaini ng with ethanol, to better characterize the heterochromatin. Metaphases were photographed under an Olympus microscope and analyzed using the Image Pro-Plus software, version 4 (Media Cybernetics, Bethesda, MD, USA). The chromosomes were measured and the centromere index (CI), relative length (RL), and centromere ratio (CR) were estimated. The chromosomes were ranked and classified according to the scheme of Green and Sessions [50].

## Abbreviations

rDNA: ribosomal DNA; DAPI: 4 6-diamidino-2-phenylindole; NOR: Nucleolar organizer region; BI: Bayesian inference.

## Competing interests

The authors declare that they have no competing interests.

## Authors’ contributions

DPB collected the morphological, chromosomal, and molecular data, and drafted the manuscript. LFT, GAV, CS, LNW, APL and JDL helped to collect and identify the specimens. LFT contributed to the analysis of external morphology. CSB helped prepare for the cytogenetic analysis. SMRP designed and coordinated the study and helped draft the manuscript. All authors have read and approved the final manuscript.

## Supplementary Material

Additional file 1 Details (species, voucher number, sample locality, and authors) of the sequences obtained in this work and from GenBank used for phylogenetic inferences. Click here for file
