# Supplementary material for: Evaluation of the taxonomic status of populations assigned to Phyllomedusa hypochondrialis (Anura, Hylidae, Phyllomedusinae) based on molecular, chromosomal, and morphological approach
Source: BMC Genet. 2013 Aug 12;14:70. doi: 10.1186/1471-2156-14-70 (PMC3751434; doi:10.1186/1471-2156-14-70)
Supplement: Additional file 1 — Details (species, voucher number, sample locality, and authors) of the sequences obtained in this work and from GenBank used for phylogenetic inferences. [file 1471-2156-14-70-S1.doc]

| **Species**  **Additional file 1:** Details (species, voucher number, sample locality, accession number and authors) of the sequences obtained in this work and from GenBank used for phylogenetic inferences. | **Voucher** | **Locality** | **12S-tRNAval-16S** | **RAG-1 (1)** | **Reference** |
| --- | --- | --- | --- | --- | --- |
| *Hylomantis hulli* | To be deposited in SMNS | Ecuador, Napo | GQ366226 | GQ366073 | Faivovich et al. 2010 |
| *Phasmahyla guttata* | CFBH 5756 | Brazil, São Paulo, Ubatuba | AY843716 | AY844489 | Faivovich et al. 2005 |
| *Phyllomedusa ayeaye* | FSFL 858 | Brazil, Minas Gerais, Congonhas do Campo | GQ366265 | — | Faivovich et al. 2010 |
| *Phyllomedusa ayeaye^1^* | FSFL 857 | Brazil, Minas Gerais, Congonhas do Campo | GQ366266 | — | Faivovich et al. 2010 |
| *Phyllomedusa ayeaye^1^* | CFBH15672 | Brazil , São Paulo, Pedregulho | GQ366243 | — | Faivovich et al. 2010 |
| *Phyllomedusa ayeaye* | CHUNB 51421 | Brazil , Minas Gerais, P. N. Serra da Canastra | GQ366244 | — | Faivovich et al. 2010 |
| *Phyllomedusa ayeaye* | CHUNB 51414 | Brazil, Minas Gerais, Poços de Caldas | GQ366245 | — | Faivovich et al. 2010 |
| *Phyllomedusa azurea* | MZUSP 70801 | Argentina, Santa Fé | GQ366247 | — | Faivovich et al. 2010 |
| *Phyllomedusa azurea* | ERW 1119 | ___ | GQ896276* | __ | Wiens et al., 2010 |
| *Phyllomedusa azurea* | CFBH 2576 | Brazil, Mato Grosso, Corumbá | GQ366248 | — | Faivovich et al. 2010 |
| *Phyllomedusa azurea* | MLP DB 3449 | Argentina, Santa Fé | GQ366249 | GQ366084 | Faivovich et al. 2010 |
| *Phyllomedusa azurea* | MLP DB 2795 | Argentina, Charata | GQ366250 | — | Faivovich et al. 2010 |
| *Phyllomedusa azurea* | MNKA9127 | Bolívia, Santa Cruz | FJ789928* | __ | Jansen et al., 2011 |
| *Phyllomedusa azurea* | MNKA9489 | Bolívia, Santa Cruz | FJ789929* | __ | Jansen et al., 2011 |
| *Phyllomedusa azurea* | MNKA9608 | Bolívia, Santa Cruz | FJ789930* | __ | Jansen et al., 2011 |
| *Phyllomedusa azurea* | Specimen not collected | Bolívia, Santa Cruz | FJ789931* | __ | Jansen et al., 2011 |
| *Phyllomedusa azurea* | Specimen not collected | Bolívia, Santa Cruz | FJ789932* | __ | Jansen et al., 2011 |
| *Phyllomedusa azurea* | MNKA9806 | Bolívia, Santa Cruz | FJ789933* | __ | Jansen et al., 2011 |
| *Phyllomedusa azurea* | ZUEC15891 | Brazil, Minas Gerais, Uberlândia | KC520762; KC520713 | KC520744 | This study |
| *Phyllomedusa azurea* | ZUEC15889 | Brazil, Minas Gerais, Uberlândia | KC52076; KC520712 | KC520743 | This study |
| *Phyllomedusa bahiana* | CFBH 2596 | Brazil , Sergipe, Areia Branca | GQ366251 | — | Faivovich et al. 2010 |
| *Phyllomedusa baltea* | To be deposited in SMNS | Peru, Pasco, Santa Cruz | GQ366252 | GQ366085 | Faivovich et al. 2010 |
| *Phyllomedusa bicolor* | AMNHA-168459 | Pet (Germany) | AY843723 | AY844495 | Faivovich et al., 2005; 2010 |
| *Phyllomedusa boliviana* | CFBH 2571 | Brazil , Mato Grosso, Corumbá | GQ366253 | GQ366086 | Faivovich et al. 2010 |
| *Phyllomedusa boliviana* | To be deposited in SMNS | Bolívia, Santa Cruz, Samaipata | GQ366254 | — | Faivovich et al. 2010 |
| *Phyllomedusa burmeisteri* | CFBHt 152 | Brazil , Espírito Santo, Linhares | GQ366255 | GQ366087 | Faivovich et al. 2010 |
| *Phyllomedusa burmeisteri* | CFBH 17360 | Brazil , Minas Gerais, Furnas | GQ366256 | — | Faivovich et al. 2010 |
| *Phyllomedusa burmeisteri* | FSFL 429 | Brazil , Minas Gerais, Varginia | GQ366257 | — | Faivovich et al. 2010 |
| *Phyllomedusa camba* | UFMT 1909 | Brazil, Mato Grosso, Vale do São Domingos | GQ366258 | GQ366088 | Faivovich et al. 2010 |
| *Phyllomedusa camba* | CFBH 17278 | Brazil , Rondônia, Ministra Andreazza | GQ366259 | — | Faivovich et al. 2010 |
| *Phyllomedusa centralis* | CHUNB 12570 | Brazil, Mato Grosso, Chapada dos Guimarães | GQ366260 | — | Faivovich et al. 2010 |
| *Phyllomedusa centralis* | CHUNB 12571 | Brazil, Mato Grosso, Chapada dos Guimarães | GQ366261 | — | Faivovich et al. 2010 |
| *Phyllomedusa distincta* | CFBH 2658 | Brazil, Paraná, Guaratiba | GQ366262 | — | Faivovich et al. 2010 |
| *Phyllomedusa distincta* | CFBH 2114 | Brazil, São Paulo, Ribeirão Branco | GQ366263 | — | Faivovich et al. 2010 |
| *Phyllomedusa hypochondrialis* | AMNH A-141109 | Guyana, Dubulay Ranch on the Berbice River | AY843724 | — | Faivovich et al., 2005; 2010 |
| *Phyllomedusa hypochondrialis* | 109AF | Brownsberg, Suriname | EU201085 | __ | Fourquet et al., 2007 |
| *Phyllomedusa hypochondrialis* | 49mc | French Guiana | EF370679 |  | Salducci et al., 2005 |
| *Phyllomedusa hypochondrialis* | ZUEC13503 | Brazil, Mato Grosso, Santa Terezinha | KC52075; KC520708 | KC520735 | This study |
| *Phyllomedusa hypochondrialis* | ZUEC15884 | Brazil, Mato Grosso, Chapada dos Guimarães | KC520733 | KC520740 | This study |
| *Phyllomedusa hypochondrialis* | ZUEC15886 | Brazil, Mato Grosso, Chapada dos Guimarães | KC520760 | KC520741 | This study |
| *Phyllomedusa hypochondrialis* | ZUEC15888 | Brazil, Mato Grosso, Chapada dos Guimarães | KC520761 | KC520742 | This study |
| *Phyllomedusa hypochondrialis* | ZUEC16194 | Brazil, Amapá, Laranjal do Jari | KC520764; KC520723 | __ | This study |
| *Phyllomedusa hypochondrialis* | ZUEC16198 | Brazil, Amapá, Laranjal do Jari | KC520714 | __ | This study |
| *Phyllomedusa hypochondrialis* | ZUEC16196 | Brazil, Amapá, Laranjal do Jari | KC520726 | __ | This study |
| *Phyllomedusa hypochondrialis* | ZUEC16505 | Brazil, Pará, Belterra | Submitted to GenBank | KC520751 | This study |
| *Phyllomedusa hypochondrialis* | ZUEC16506 | Brazil, Pará, Belterra | Submitted to GenBank | KC520751 | This study |
| *Phyllomedusa hypochondrialis* | ZUEC16511 | Brazil, Pará, Prainha | KC520721 | KC520753 | This study |
| *Phyllomedusa hypochondrialis* | ZUEC16512 | Brazil, Pará, Prainha | KC520722 | KC520754 | This study |
| *Phyllomedusa hypochondrialis* | ZUEC19921 | Brazil, Pará, Óbidos | KC520728 | __ | This study |
| *Phyllomedusa hypochondrialis* | ZUEC19914 | Brazil, Pará, Óbidos | KC520706 | __ | This study |
| *Phyllomedusa hypochondrialis* | ZUEC19915 | Brazil, Pará, Óbidos | KC520716 | __ | This study |
| *Phyllomedusa hypochondrialis* | ZUEC19916 | Brazil, Pará, Monte Alegre | KC520732 | __ | This study |
| *Phyllomedusa hypochondrialis* | ZUEC19922 | Brazil, Pará, Oriximiná | KC520729 | __ | This study |
| *Phyllomedusa hypochondrialis* | ZUEC 19926 | Brazil, Pará, Oriximiná | Submitted to GenBank |  | This study |
| *Phyllomedusa hypochondrialis* | ZUEC19930 | Brazil, Pará, Alenquer | KC520730 | __ | This study |
| *Phyllomedusa hypochondrialis* | ZUEC19932 | Brazil, Amapá, Alenquer | KC520731 | __ | This study |
| *Phyllomedusa hypochondrialis* | ZUEC19917 | Brazil, Pará, Alenquer | KC520727 | __ | This study |
| *Phyllomedusa hypochondrialis* | ZUEC19942 | Brazil, Pará, Alenquer | KC520702 | __ | This study |
| *Phyllomedusa sp.* (aff. *hypochondrialis)* | ZUEC13486 | Brazil, Tocantins, Porto Nacional | KC52075; KC520703 | KC520748 | This study |
| *Phyllomedusa sp.* (aff. *hypochondrialis)* | ZUEC13491 | Brazil, Tocantins, Porto Nacional | KC520704 | KC520745 | This study |
| *Phyllomedusa sp.* (aff. *hypochondrialis)* | ZUEC13492 | Brazil, Tocantins, Porto Nacional | KC520707 | __ | This study |
| *Phyllomedusa sp.* (aff. *hypochondrialis)* | ZUEC13662 | Brazil, Maranhão, Urbano Santos | KC520709 | KC520736 | This study |
| *Phyllomedusa sp.* (aff. *hypochondrialis)* | ZUEC13665 | Brazil, Maranhão, Urbano Santos | KC520710 | KC520738 | This study |
| *Phyllomedusa sp.* (aff. *hypochondrialis)* | ZUEC13663 | Brazil, Maranhão, Urbano Santos | KC520759; KC520715 | KC520737 | This study |
| *Phyllomedusa sp.* (aff. *hypochondrialis)* | ZUEC16221 | Brazil, Maranhão, Bacabeira | KC52076; KC520711 | KC520747 | This study |
| *Phyllomedusa sp.* (aff. *hypochondrialis)* | ZUEC16225 | Brazil, Maranhão, Bacabeira | KC520767 | __ | This study |
| *Phyllomedusa sp.* (aff. *hypochondrialis)* | ZUEC16226 | Brazil, Maranhão, Bacabeira | KC520718 | KC520749 | This study |
| *Phyllomedusa sp.* (aff. *hypochondrialis)* | ZUEC16237 | Brazil, Maranhão, Bacabeira | KC520768; KC520719 | KC520750 | This study |
| *Phyllomedusa sp.* (aff. *hypochondrialis)* | ZUEC16212 | Brazil, Maranhão, São Luis | KC520765 | KC520746 | This study |
| *Phyllomedusa sp.* (aff. *hypochondrialis)* | ZUEC16216 | Brazil, Maranhão, São Luis | KC520717 | __ | This study |
| *Phyllomedusa sp.* (aff. *hypochondrialis)* | ZUEC17071 | Brazil, Bahia, Barreiras | KC520724 | __ | This study |
| *Phyllomedusa sp.* (aff. *hypochondrialis)* | ZUEC17072 | Brazil, Bahia, Barreiras | KC520725 | __ | This study |
| *Phyllomedusa* cf. *hypochondrialis* | ZUEC16406 | Brazil, Mato Grosso, Alta Floresta | KC520759; KC520720 | __ | This study |
| *Phyllomedusa iheringii* | MNRJ 18782 | Brazil, Rio Grande do Sul, Santa Maria | GQ366264 | — | Faivovich et al. 2010 |
| *Phyllomedusa megacephala* | MCNAM 6339 | Brazil, Minas Gerais, Serra do Cipó | GQ366267 | GQ366089 | Faivovich et al. 2010 |
| *Phyllomedusa megacephala* | MCNAM 6338 | Brazil, Minas Gerais, Santana do Riacho | GQ366268 | — | Faivovich et al. 2010 |
| *Phyllomedusa megacephala* | CFBH 10225 | Brazil, Minas Gerais, Grão Mongol | GQ366269 | GQ366090 | Faivovich et al. 2010 |
| *Phyllomedusa neildi* | CVULA 6503 | Venezuela, Falcon, Municipio Petit | GQ366270 | — | Faivovich et al. 2010 |
| *Phyllomedusa nordestina* | CFBH 7330 | Brazil, Alagoas, Passos dos Camarajibe | GQ366271 | GQ366091 | Faivovich et al. 2010 |
| *Phyllomedusa nordestina* | CHUNB 44443 | Brazil, Minas Gerais, Butiazeiro | GQ366272 | — | Faivovich et al. 2010 |
| *Phyllomedusa oreades^2^* | CHUNB 56879 | Brazil, Minas Gerais, Perdizes | GQ366241 | — | Faivovich et al. 2010 |
| *Phyllomedusa oreades* | CHUNB 56871 | Brazil, Goiás, Serra de Caldas | GQ366275 | — | Faivovich et al. 2010 |
| *Phyllomedusa oreades* | CHUNB 51424 | Brazil, Brasília | GQ366276 | — | Faivovich et al. 2010 |
| *Phyllomedusa oreades* | CHUNB 56875 | Brazil, Goiás, Serra de Caldas | GQ366278 | — | Faivovich et al. 2010 |
| *Phyllomedusa oreades* | CHUNB 49937 | Brazil, Goiás, Minacu | GQ366279 | — | Faivovich et al. 2010 |
| *Phyllomedusa palliata* | To be deposited in SMNS | Bolívia, Beni, Rurrenabaque | GQ366280 | GQ366092 | Faivovich et al. 2010 |
| *Phyllomedusa palliata* | WED55638 | Peru, Madre de Dios | AY326046 | __ | Darst and Canatella, 2004 |
| *Phyllomedusa perinesos* | KU178854 | Ecuador, Napo | GQ896278 | __ | Wiens et al., 2010 |
| *Phyllomedusa “rohdei”* | CFBHt 93 | Brazil, São Paulo, Ubatuba | GQ366237 | GQ366080 | Faivovich et al. 2010 |
| *Phyllomedusa “rohdei”* | CFBH 7196 | Brazil, São Paulo, Santo Antônio do Pinhal | GQ366238 | GQ366081 | Faivovich et al. 2010 |
| *Phyllomedusa “rohdei”* | MNRJ 40691 | Brazil, Espírito Santo, Santa Tereza | GQ366239 | — | Faivovich et al. 2010 |
| *Phyllomedusa “rohdei”* | CRR-18 | Brazil, Minas Gerais, Perdizes | GQ366240 | GQ366082 | Faivovich et al. 2010 |
| *Phyllomedusa sauvagii* | CFBH 2573 | Brazil, Mato Grosso, Corumbá | GQ366281 | GQ366093 | Faivovich et al. 2010 |
| *Phyllomedusa sauvagii* | MACN 40002 | Argentina, Salta | GQ366282 | GQ366094 | Faivovich et al. 2010 |
| *Phyllomedusa sauvagii* | CFBH 14250 | Brazil, Mato Grosso, Bonito | GQ366283 | — | Faivovich et al. 2010 |
| *Phyllomedusa tarsius* | MJH 67 | Brazil, Amazonas, Reserva Ducke | AY843726 | GQ366095 | Faivovich et al., 2005; 2010 |
| *Phyllomedusa tetraploidea* | CFBH 2464 | Brazil, São Paulo, Ribeirão Branco | GQ366284 | GQ366096 | Faivovich et al. 2010 |
| *Phyllomedusa tetraploidea* | CFBH 1725 | Brazil, São Paulo, Ribeirão Branco | GQ366285 | — | Faivovich et al. 2010 |
| *Phyllomedusa tetraploidea* | MACN 37796 | Argentina, Misiones | AY843727 | — | Faivovich et al., 2005 |
| *Phyllomedusa tomopterna* | CFBH 2451 | Amazonas, Manaus | GQ366286 | — | Faivovich et al. 2010 |
| *Phyllomedusa tomopterna* | MJH 7076 | Peru, Huanuco | AY843728 | AY844497 | Faivovich et al., 2005; 2010 |
| *Phyllomedusa tomopterna* | KU205428 | Peru, Madre de Dios | AY326045.1 | __ | Darst and Canatella, 2004 |
| *Phyllomedusa trinitatis* | CVULA7086 | Venezuela, Miranda | GQ366287 | GQ366097 | Faivovich et al. 2010 |
| *Phyllomedusa vaillanti* | AMNH A- 166288 | Guyana, Berbice River | AY549363 | AY844498 | Faivovich et al., 2005 |
| *Phyllomedusa sp.* | CAUC0761 | Brazil:Santa Catarina: Água Doce | KC520734 | KC520755 | This study |
| *Phyllomedusa sp.* | CAUC0762 | Brazil:Santa Catarina: Água Doce | KC520705 | KC520756 | This study |

Taxonomic names according to Frost (2013). ^1^ Specimens deposited in Genbank as *P. araguari*, ^2^ Specimens deposited in Genbank as *P. itacolomi*, *Small fragment isolate of mitochondrial 16S ribosomal RNA gene (~540bp). Abreviation: AMNH (American Museum of Natural History, USA); CAUC (Coleção Anfíbios Unochapecó, Brazil), CFBH (Coleção Célio Fernando Baptista Haddad, Universidade Estadual Paulista, Brazil); CHUNB (Coleção Herpetológica Universidade Nacional de Brasília, Brazil); CRR (Camila R. Rabelo Field Series); CVULA (Colección de Vertebrados Universidad de los Andes, Venezuela); FSFL (Felipe Sá Fortes Leite and Bruno Pachecco field series); KU (The University of Kansas, Museum of Natural History, Lawrence, Kansas, USA); MACN (Museo Argentino de Ciencias Naturales ‘‘Bernardino Rivadavia’’, Argentina); MCNAM (Museu de Ciências Naturais, Pontifícia Universidade Católica de Minas Gerais, Brazil); MJH (Martin J. Henzel field series); MLP DB (Colección Herpetología Diego Baldo, Museo de La Plata, Argentina) MNRJ (Museu Nacional Universidade Federal do Rio de Janeiro, Brazil); UFMT (Universidade Federal do Mato Grosso, Brazil); ZUEC (Museu de Zoologia “Prof. Dr. Adão José Cardoso”, Brazil).

**Reference**

Darst CR, Cannatella DC: **Novel relationships among hyloid frogs inferred from 12S and 16S mitochondrial DNA sequences.** Mol. Phylogen Evol. 2004, 31: 462–475.

Faivovich J, Haddad CFB, Garcia PCA, Frost DR, Campbell JA, Wheeler WC: **A systematics review of the frog family Hylidae, with special reference to the Hylinae, a phylogenetic analysis and taxonomic revision**. Bul. Am. Nat. Hist. 2005, 294: 1-240.

Faivovich J, Haddad CFB, Baêta D, Jungfer KH, Álvares GFRA, Brandão RA, Sheil C, Barrientos LS, Barrio-Amós CL, Cruz CAG, Wheeler WC: **The phylogenetic relationships of the charismatic poster frogs, Phyllomedusinae (Anura, Hylidae).** Cladistics 2010, 25: 1-35.

Fouquet A, Gilles A, Vences M, Marty C, Blanc M, Gemmell NJ: **Underestimation of species richness in Neotropical frogs revealed by mtDNA analyses**. Plos One 2007, 2(10) (e1109): 1101-1110

Frost DR: **Amphibian Species of the World: An online reference.** [http://research.amnh.org/vz/herpetology/amphibia/].

Jansen M, Bloch R, Schulze A, Pfenninger M: **Integrative inventory of Bolivia’s lowland anurans reveals hidden diversity**. Zoologica Scripta 2011, 40:567–583.

Wiens JJ, Kuczynski CA, Hua X, Moen DC: **An expanded phylogeny of treefrogs (Hylidae) based on nuclear and mitochondrial sequence data.** Mol. Phylogen. Evol. 2010, 55: 871–882.
